# Recurrent neural network-based acute concussion classifier using raw resting state EEG data

**DOI:** 10.1038/s41598-021-91614-4

**Published:** 2021-06-11

**Authors:** Karun Thanjavur, Arif Babul, Brandon Foran, Maya Bielecki, Adam Gilchrist, Dionissios T. Hristopulos, Leyla R. Brucar, Naznin Virji-Babul

**Affiliations:** 1 Department of Physics and Astronomy, University of Victoria, Victoria, BC V8P 5C2 Canada; 2 Department of Computer Science, Middlesex College, Western University, London, ON N6A 5B7 Canada; 3 School of Electrical and Computer Engineering, Technical University of Crete, 73100 Chania, Greece; 4 Djavad Mowafaghian Centre for Brain Health, University of British Columbia, Vancouver, BC V6T 1Z3 Canada; 5 Department of Physical Therapy, Faculty of Medicine, University of British Columbia, Vancouver, BC V6T 1Z3 Canada

**Keywords:** Neuroscience, Brain injuries, Machine learning

## Abstract

Concussion is a global health concern. Despite its high prevalence, a sound understanding of the mechanisms underlying this type of diffuse brain injury remains elusive. It is, however, well established that concussions cause significant functional deficits; that children and youths are disproportionately affected and have longer recovery time than adults; and that individuals suffering from a concussion are more prone to experience additional concussions, with each successive injury increasing the risk of long term neurological and mental health complications. Currently, the most significant challenge in concussion management is the lack of objective, clinically- accepted, brain-based approaches for determining whether an athlete has suffered a concussion. Here, we report on our efforts to address this challenge. Specifically, we introduce a deep learning long short-term memory (LSTM)-based recurrent neural network that is able to distinguish between non-concussed and acute post-concussed adolescent athletes using only short (i.e. 90 s long) samples of resting state EEG data as input. The athletes were neither required to perform a specific task nor expected to respond to a stimulus during data collection. The acquired EEG data were neither filtered, cleaned of artefacts, nor subjected to explicit feature extraction. The LSTM network was trained and validated using data from 27 male, adolescent athletes with sports related concussion, benchmarked against 35 non-concussed adolescent athletes. During rigorous testing, the classifier consistently identified concussions with an accuracy of > 90% and achieved an ensemble median Area Under the Receiver Operating Characteristic Curve (ROC/AUC) equal to 0.971. This is the first instance of a high-performing classifier that relies only on easy-to-acquire resting state, raw EEG data. Our concussion classifier represents a promising first step towards the development of an easy-to-use, objective, brain-based, automatic classification of concussion at an individual level.

## Introduction

Concussion or mild traumatic brain injury (mTBI) is an urgent public health concern. Canadian^1^ and US data^2,3^ indicate an annual rate of reported mTBIs of 1100 per 100,000 people, 75% of which involve children, youths and young adults^4^. The actual rate is likely much higher as individuals suffering from concussion often do not seek medical advice^5,6^. Despite its high prevalence, a sound understanding of the mechanisms underlying mTBI remains elusive. What is clear is that concussions induce diffuse, heterogeneous, spatially distributed changes in brain structure and function, resulting in cognitive, motor, emotional, and behavioural challenges that can persist for many months^7–9^. Children and youths are especially vulnerable: they are disproportionately affected by concussions^10^ and take longer than adults to recover^7,11^. Moreover, the effects of concussion on their developing brain can lead to compromised brain and mental health, impairing learning, working, and socializing^12^. Such disruptions at this critical developmental period in the lives of children and youths can be devastating.

The management of concussion suffers from a key challenge: there is no accepted, objective, brain-based tool to make an initial diagnosis of concussion. The current gold standard involves a clinical assessment by an experienced physician in accordance with the criteria outlined in the Berlin consensus statement^13^. This approach is difficult to standardize as it is dependent on the individual physician and relies on subjective reporting of the symptoms. Symptoms, however, do not appear to be directly correlated to the pathophysiological mechanisms responsible for the cognitive, emotional and behavioural deficits^14,15^, and several studies^15–24^ have shown that structural and functional abnormalities in the brain persist even after symptom resolution, especially in pediatric patients. Manning et al.^21^ and Hristopulos et al.^25^ hypothesize that the increased vulnerability of children and youths to brain injury is a consequence of the effects of concussion being overlaid on brains whose structural and functional organization are undergoing dynamic changes due to development. Studies also show that children and youths who have sustained a concussion are at a higher risk of sustaining additional concussions, which can then lead to neuro-psychiatric and neuro-degenerative disorders^26,27^.

Numerous groups have sought to leverage neuroimaging data to detect mTBIs reliably and objectively. At the group-level, structural changes in the integrity of white matter in adolescents have been observed using diffusion tensor imaging (DTI)^21,28–33^. Similarly, studies of youths during “resting state” reveal significant mTBI-induced alterations in the functional organization of the brain with respect to their non-concussed counterparts, with key features being (1) an increase in the beta-band power (30–45 Hz)^34^, (2) an increase in functional connectivity (hyperconnectivity)^18,35,36^, and (3) disrupted information flow patterns^25^, again particularly in the frontal regions. All of these studies rely on summary measures to characterize functional changes in the brain. However, because the brain injury due to mTBI is diffuse and the resulting changes in the brain structure and function are subtle, the summary measures cannot be used to statistically differentiate between non-concussed and injured at the *individual* level. This has led the Radiological Society of North America to advise that “in pediatric patients, imaging should not be routinely obtained to diagnose mild TBI” because “there is insufficient evidence [to support] the routine clinical use of advanced neuroimaging techniques for diagnosis and/or prognostication at the individual patient level”^37^.

Rapid advances in machine learning (ML) and artificial intelligence (AI) methods have opened up new ways to explore neuroimaging data^38–41^. ML includes methods of data analysis that automate pattern recognition and model building. ML systems learn to identify relevant, often latent, features directly from the data. Consequently, they are especially suited for analysis of complex, high-dimensional datasets that themselves are products of often poorly understood dynamics. Focusing specifically on mTBI, several groups have attempted to develop ML algorithms to detect concussions. Examples of such attempts include the use of support vector machine (SVM) to distinguish between non-concussed (hereafter referred to as “control”) and concussed individuals using explicit features derived from resting state magnetoencephalography (MEG) data^42^ or resting state electroencephalography (EEG) recordings^43^; the use of random forest^44^ and SVM^45^ to classify participants on the basis of structural connectivity features derived from diffusion magnetic resonance imaging (dMRI) data, with Vergara et al.^45^ augmenting the dMRI measures with functional magnetic resonance imaging (fMRI)-based resting state functional network connectivity measures; the use of SVM to classify young adults using features from multiple task-related EEG recordings^46^; the use of an ensemble of shallow multilayer perceptron network with one hidden layer to distinguish between control and concussed adolescents using descriptors derived from resting state EEG data^47^; and most recently, the use of a deep learning convolution neural network to classify adolescents using EEG recordings of single-trial event-related potentials^48^.

The goal of our research is to develop a reliable, accurate, easy-to-use deep learning neural network-based classifier (hereafter, *Conc*ussion classification *Net*work or *ConcNet*) that can distinguish between control and acute post-concussed individuals using only a *short* (90 s long, in our case) sample of *raw*, *resting state EEG* data (hereafter, raw90-rsEEG) as input. Our approach differs from those of other ML-based efforts in four notable ways: (1) We exclusively use EEG data as input for our classifier; specifically, we do not augment the EEG data with any clinical measures. (2) We only work with resting state EEG data; that is, EEG recordings collected in the absence of an imposed stimulus. The resulting signals reflect the spontaneous ongoing background neuronal activity in the brain^49^. (3) Our classifier receives, as input, the actual recorded time-series data, not a set of features derived from the data. (4) Finally, the input is, in fact, the “raw” time-series, where “raw” refers to data that have neither been cleaned to remove known artefacts, like eye blinks and motion artefacts, nor filtered to remove high or low frequencies.

Our decision to select EEG as the modality of choice was informed by a number of considerations, including the fact that (1) it is an inexpensive, non-invasive, direct, fine-time resolution probe of the neural activity in individuals; (2) at the group-level, it can reliably and objectively detect functional changes in the brain due to mTBI^25,29,50,51^; and (3) it does not require elaborate infrastructure, the introduction of contrast agents and radioisotopes, or exposure to radiation. Additionally, resting state data are easy to collect; the participant is neither required to perform a specific task nor subjected to a stimulus.

One challenge of working with EEG time series, particularly resting state EEG signals, is that they consist not only of electrical signals from the brain, but also a variety of contaminants and distortions from the environment (e.g. strong power line interference at 60 Hz) and physiological sources (e.g. electrical signals generated by cardiac activity, by nerves responsible for innervating the muscles, and by muscle activity associated with eye blinks as well as eye and head motion). Typically, the acquired EEG data are filtered and cleaned to remove these artefacts (c.f. Rotem-Kohavi et al.^52,53^, Munia et al.^20^), with cleaning strategies in use ranging from fully automated algorithms to mixed schemes where some artefacts are removed via automated algorithms and others are manually identified and removed.

The lack of a standardized, consensus cleaning pipeline and more importantly, the lack of well-defined measures for quantifying a dataset’s degree of “cleanliness” pose a serious challenge^54–56^. Additionally, commonly used artefact removal schemes are designed to treat known distortions, leaving open the possibility that the data may still be contaminated by latent artefacts. Even in the case of known artefacts, the identification-decontamination strategies are based on specific assumptions about the morphology of the distortions and the manner in which they interact with the signals of interest. These do not always fully capture the diverse ways in which the artefacts manifest in real recordings^55,57^, and improper cleaning can introduce new distortions and unknown biases^55,58^. Meisler et al.^59^ assert that commonly used data cleaning methods do not necessarily improve brain state classification. In deliberately choosing to work with *raw* data, we circumvent these concerns and instead rely on the deep learning network to learn the relevant aspects of the data while ignoring the rest. In the process, we also avoid having to grapple with the complex problem of feature selection^60–62^, which itself can be a source of bias^61,63,64^, as well as confounding effects on the feature values due to the EEG signals’ strongly non-stationary character in both the temporal and the spectral domains^65–67^.

In designing our classifier, we have adopted a recurrent neural network architecture comprising two layers of bi-directional long short-term memory (LSTM)^68,69^ units. An LSTM-based network is ideally suited for processing an ordered sequence of data points, such as the EEG time series data, where there is a high likelihood of temporal correlations and/or causal relationships between measurements at different time points and where the time lag between these could potentially be large^70^. For a more detailed exposition of the reasons informing our decision to adopt a bi-directional LSTM network, including recent findings from the studies of brain-computer interface systems, we refer the reader to “Methods” section. As for training, validating and testing the network, we used a total of 216 EEG samples obtained from 27 male adolescent athletes with sports related concussion within 1 month of injury, who met the concussion diagnostic criteria consistent with the Berlin consensus statement^13^, and 280 samples from 35 age-matched control athletes. For the concussed participants, the date and time of the direct blow was documented by the team coach as per the consensus statement, and the team physician or an experienced physician with expertise in concussions made the diagnosis of concussion based on the Berlin consensus statement. The number of symptoms and symptom severity of each concussed participant were assessed using either the Sports Concussion Assessment Tool 3 (SCAT3) or the Child Sports Concussion Assessment Tool 3 (Child SCAT3) both at the time of diagnosis as well as at the time of data collections (see “Methods” section for full details).

## Results

In Table 1, we present the demographic information about the participants in this study. All of the concussed participants met the Berlin criteria and exhibited from 4 to 22 SCAT3 symptoms, at the time of testing. The symptoms most frequently reported included irritability, sensitivity to light, dizziness, fatigue, “don’t feel right”, and difficulty concentrating/remembering.

**Table 1:** Demographic information for the participants involved in this study. SD stands for standard deviation.

Demographic information	Non-concussed (control)	Concussed
Age in years (SD)	14.7 (2.1)	13.5 (2.6)
Sex	100% male	100% male
Time since concussion		100% within one month
SCAT: Number of symptoms (SD)		9.2 (6.2)
SCAT: Symptom severity (SD)		21.7 (18.7)
Child SCAT: Number of symptoms (SD)		13.0 (5.4)
Child SCAT: Symptom severity (SD)		23.2 (13.6)

Most previous EEG resting state studies consider filtered and cleaned data and the corresponding power spectra have been shown in various papers (c.f. Figure 2 of Balkan et al.^34^ or Figure 1 of Ivarsson et al.^71^). In this study we work with raw data. In the Supplementary section, we show the power spectra of the raw resting state EEG signal from six regions of the scalp for both the control and concussed groups (see Figures. S1 and S2). A cursory visual inspection shows that the power spectra for the two groups are broadly similar. In detail, there are small differences in the median power at specific frequencies between the two groups; however, there is significant scatter in the individual power spectra around the median in both groups. We briefly comment on the potential relevance of the differences in the context of previous efforts to design feature-based classifiers in the Supplementary section.

The development and assessment of *ConcNet*   proceeded in three stages. Stage 1 is the *exploratory phase* during which we establish the suitability of *ConcNet* without attempting to optimize it. Stage 2 focuses on optimizing *ConcNet* and estimating the classification accuracy of the resulting network. Finally, Stage 3 focuses on the statistical assessment of *ConcNet* ’s classification performance. Here, we briefly summarize these three stages, focusing on the results. Additional details are provided in the “Methods” section.

### Stage 1: Identifying a promising machine learning algorithm for *ConcNet*

A number of different ML techniques have been proposed to tackle classification problems (see Kotsiantis et al.^72^ and Singh et al.^73^ for comprehensive reviews). Prior to settling on a deep learning neural network-based classifier, and specifically an LSTM-based deep learning recurrent neural network, we experimented with other options, including using a SVM to distinguish between control and concussed individuals on the basis of features derived from filtered and cleaned resting state EEG data^43^, and using an ensemble of multilayer perceptron networks to distinguish between the two populations on the basis of descriptors derived from filtered but not cleaned resting state data^47^.

None of our previous efforts proved satisfactory, with the features-based algorithms achieving an *Accuracy* of no more than $65\%$ during testing. (*Accuracy*  is the fraction of classifications that are correct, a commonly used assessment metric; for convenience, we recall the definition of the standard performance metrics we use in our work in Table 2.) Since our aim is to develop a clinically viable classifier, we considered an algorithm as promising only if its *Accuracy*  is $>80\%$ during the Stage 1 exploratory phase. The rationale behind this particular threshold criterion is explained in “Methods” section (“Network training, validation and testing” section). Our LSTM-based classifier, the focus of this paper, achieved an *Accuracy* score of $88.9\%$.

**Table 2:** Common measures used in this paper to gauge the performance of classification tasks. In the formulae above, TP (True Positives) is the number of correctly classified concussed individuals; TN (True Negatives) is the number of correctly classified control participants; while FP and FN are the numbers of falsely classified control (i.e. False Positives) and concussed (i.e. False Negatives) participants, respectively.

Performance Metric	Definition	Alternate name	
*Accuracy*	$\frac{\mathrm{TP}+\mathrm{TN}}{\mathrm{TP}+\mathrm{TN}+\mathrm{FP}+\mathrm{FN}}$		
*Recall*	$\frac{\mathrm{TP}}{\mathrm{TP}+\mathrm{FN}}$	also True Positive Rate (*TPR*)	
*Precision*	$\frac{\mathrm{TP}}{\mathrm{TP}+\mathrm{FP}}$	also Positive Predictive Value (*PPV*)	
*Specificity*	$\frac{\mathrm{TN}}{\mathrm{TN}+\mathrm{FP}}$	also True Negative Rate (*TNR*)	
*Miss Rate*	$\frac{\mathrm{FN}}{\mathrm{FN}+\mathrm{TP}}$	also False Negative Rate (*FNR*)	$FNR\equiv 1-TPR$
False Discovery Rate (*FDR*)	$\frac{\mathrm{FP}}{\mathrm{FP}+\mathrm{TP}}$		$FDR \equiv 1-PPV$
False Positive Rate (*FPR*)	$\frac{\mathrm{FP}}{\mathrm{FP}+\mathrm{TN}}$		$FPR \equiv 1-TNR$
Negative Predictive Value (*NPV*)	$\frac{\mathrm{TN}}{\mathrm{FN}+\mathrm{TN}}$		
*Informedness*	$TPR+TNR-1$		
*Markedness*	$PPV+NPV-1$		

We iterated towards our final configuration of *ConcNet*  in two stages: the initial exploratory phase and the optimization phase. The present (first stage) involved selecting the initial architecture of our LSTM network, and then training, validating and testing the network using standard protocols. Below, we focus mainly on the results. We document and explain the approach in greater detail in “Methods” section.

**Figure 1:**
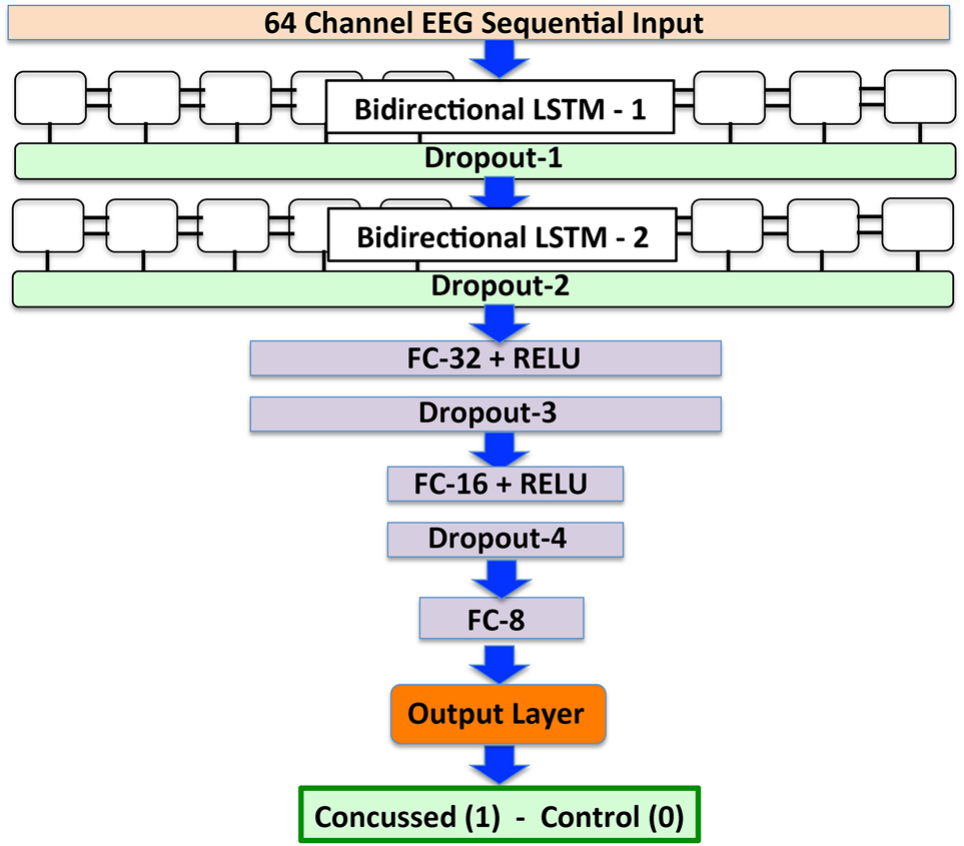
The architecture of our first generation *ConcNet*, a recurrent neural network architecture comprising two LSTM layers. The 64-channel EEG input is fed through a sequential input layer to the first recurrent layer with 100 bi-directional LSTM units. The output is then passed to a dropout layer for regularization to reduce overfitting, and then to the second LSTM layer, also with 100 bi-directional LSTM units. The output of the 100 LSTM units then passes through another dropout layer, followed by two fully connected and two dropout layers organized as shown. These two fully connected layers have 32 and 16 nodes, and use Rectified Linear Unit activation functions. The results are then fed to a third fully connected layer with 8 nodes. This layer computes the weighted sum of the inputs and passes the output to the final output layer, which uses a logistic function to assign the input raw90-rsEEG data a score ($0 \le P_{\mathrm{mTBI}} \le 1$). We classify it as *concussed* or *control* on the basis of the score. (*Flow chart created with PowerPoint v14.5.6*).

The architecture of our first generation network is shown in Fig. 1. It comprises a sequential input layer that receives the 64-channel raw90-rsEEG data, followed by two *bi-directional LSTM* layers, each with 100 memory units. These are followed by three *Fully Connected* (FC) layers with 32, 16 and 8 nodes. Each of the nodes computes the weighted sum of all the outputs from the preceding layer and adds a bias. In the case of the first two FC layers, the weighted sum is operated on by a *Rectified Linear Unit* activation function, and the result is fed to the next layer. The outputs from the nodes of the third FC layer are passed directly to the output layer which computes a linear combination of the inputs, applies a *logistic function* to the result, and assigns a *score* ($0 \le P_{\mathrm{mTBI}} \le 1$). By comparing this score to a user-chosen classification threshold value (=0.5 in our case), the original inputted raw90-rsEEG is classified as *control* (class = 0) or *concussed* (class = 1). We use the *Dropout* regularization technique to minimize overfitting by the network; consequently, there are *Dropout* layers after each of the LSTM and two of the three FC layers. Brief descriptions of the standard ML terms used here (highlighted in italics) are given in “Methods” section, while more details can be found in standard textbooks, e.g.^74^.

The training of the network was carried out using all eight of the raw90-rsEEG data samples from 19 (of 27) randomly selected concussed and 25 (of 35) randomly selected control participants, and we focused on tuning the network’s parameters (i.e. its weights and biases). We did not attempt to optimize the network’s performance or to systematically tune its hyperparameters other than to minimally adjust the learning rate.

**Figure 2:**
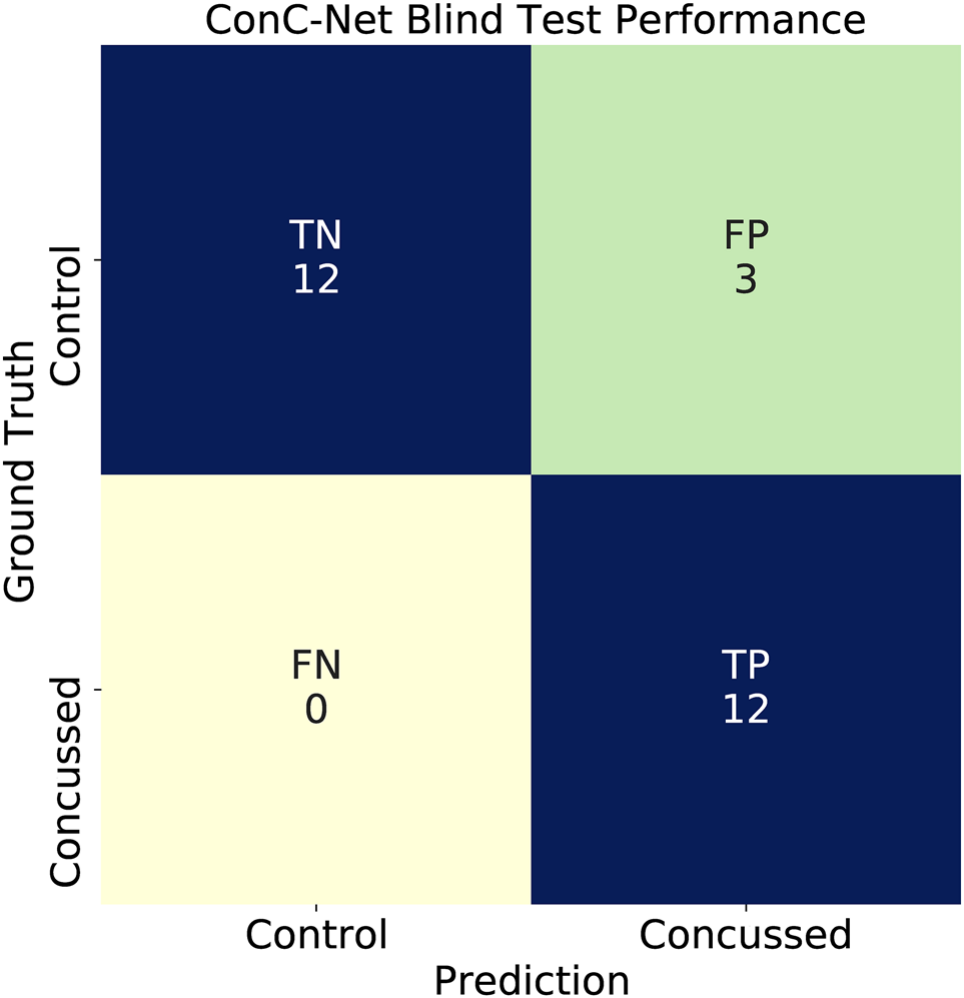
Confusion Matrix summarizing the results from the second test carried out in Stage 1, using 27 raw90-rsEEG samples drawn randomly from the 8 concussed and 10 control participants whose data had been reserved for this purpose. (*Plotted with Matplotlib v3.2.1 *^75^).

Once trained, the network (hereafter, *ConcNet*  1) was tested using 27 raw90-rsEEG samples drawn randomly from the 8 concussed and 10 control participants whose data had been held back. We emphasize that neither this particular test data set nor, for that matter, any of the data from the contributing test participants had been used in the network development up to this point. A detailed tabulation of *ConcNet*  1’s performance is shown in Table 3, and the *confusion matrix* summarizing the results is presented in Fig. 2.

**Table 3:** Details of the Stage 1 test results for *ConcNet*. The table shows the number of distinct segments from any one participant that were present in the blind test sample, and how each of these were classified. All except one pair were consistently—and correctly—classified. Final column gives the results in terms of the number of True Negative (TN), False Positive (FP), False Negative (FN) and True Positive (TP). These are further summarized in the Confusion Matrix in Fig. 2.

Blind Test / Participant	Actual Status	Segment 1	Segment 2	Segment 3	Result			
					TN	FP	FN	TP
Test 1	Concussed	$\checkmark$	$\checkmark$	$\checkmark$				3
Test 2	Control	$\checkmark$	$\checkmark$	$\checkmark$	3			
Test 3	Control	$\checkmark$			1			
Test 4	Control	$\checkmark$	$\checkmark$		2			
Test 5	Control	$\checkmark$			1			
Test 6	Control			$\checkmark$	1			
Test 7	Concussed		$\checkmark$					1
Test 8	Control	$\checkmark$	$\checkmark$		2			
Test 9	Concussed	$\checkmark$		$\checkmark$				2
Test 10	Control	$\checkmark$			1			
Test 11	Concussed		$\checkmark$	$\checkmark$				2
Test 12	Control	$\checkmark$	$\times$		1	1		
Test 13	Concussed	$\checkmark$		$\checkmark$				2
Test 14	Control	$\times$				1		
Test 15	Concussed			$\checkmark$				1
Test 16	Control	$\times$				1		
Test 17	Concussed			$\checkmark$				1
				Total:	12	3	0	12

With 24 of the 27 test samples correctly classified, *ConcNet*  1 achieved an *Accuracy* of 88.9%, which was well above our threshold for deeming an approach promising. The network correctly classified all of the concussed samples, thus achieving 100% *Recall* (also termed *Sensitivity* or True Positive Rate). Recall is a measure of the classifier’s efficacy in classifying concussed as such. However, three raw90-rsEEG samples contributed by three different control participants were misclassified, resulting in $Precision=80\%$. In this regard, the classifier worked as desired of a conservative clinical classifier: it correctly identified all the concussed participants (i.e. high recall), and misclassified only a small number of control participants.

We also note that our test dataset included several pairs of samples which had been drawn from the same participant, as shown in Table 3; in other words, the test data comprised of more than one (but not identical) raw90-rsEEG samples from some of the participants. We refer to two samples from the same participant as a *segment pair*. The motivation for including such segment pairs was to investigate if both samples of each pair would be similarly and *correctly* classified. In order to quantify this performance, we define the classifier’s *Consistency* as the ratio of the distinct number of segment pairs which are similarly and correctly classified to the total number of segment pairs. Our test sample included a total of 12 such pairs of raw90-rsEEG recordings. As shown in the results tabulated in Table 3, *ConcNet*  1 classified 11 segment pairs identically (and correctly) for a *Consistency* rating of 91.7%.

### Stage 2: Optimizing the LSTM-based *ConcNet*

Encouraged by the Stage 1 results for the LSTM design, we proceeded to systematically tune the network’s hyperparameters and internal parameters to optimize its performance. We adopted a grid search approach for tuning the principal hyperparameters, such as the number of LSTM layers and units, the number of FC layers as well as the number of nodes in each, the number of *Dropout* layers and the associated dropout fraction, and the initial learning rate. We also performed a coarse grid search to determine the optimal mini-batch size and the number of epochs used for training. A more detailed description of these latter hyperparameters and their role in training is given in Section 4.6.

**Figure 3:**
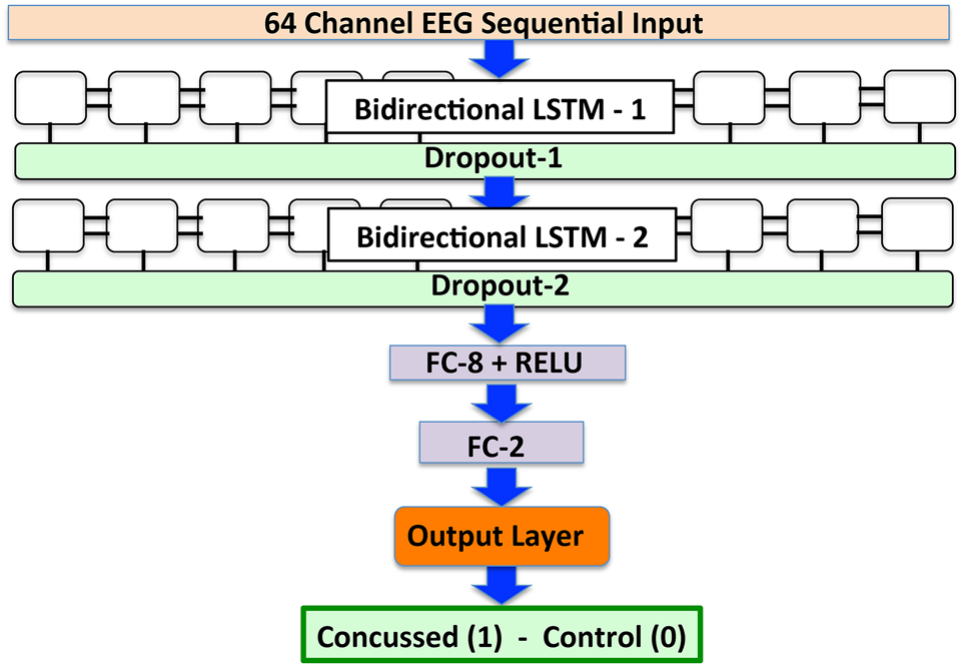
The architecture of our final streamlined version of *ConcNet*  2.0, following the systematic hyperparameter tuning in Stage 2. The architecture is similar to that shown in Fig. 1, except that this version has only 2 fully connected layers, with 8 and 2 units, and no associated dropout layers. (*Flow chart created with PowerPoint v14.5.6*).

During this stage, we used the full available dataset comprising 216 EEG samples obtained from 27 concussed athletes and 280 samples from 35 age-matched control athletes, which we partitioned into training, validation and test sets as per the 80:10:10 rule commonly used in Machine Learning. (See “Methods” section for further details.) The final network architecture of *ConcNet* 2, shown in Fig. 3, is more streamlined than *ConcNet*  1. It has one less FC layer and fewer nodes per FC layer. With the reduction of nodes in the remaining FC layers, the *Dropout* layers present after each FC layer in *ConcNet*  1 (c.f. Fig. 1) were no longer necessary.

The more streamlined *ConcNet*  2 also formally fared a bit better than *ConcNet*  1 in that the number of false positives dropped from 3 to 2; correspondingly, the *Accuracy* increased to 92.6%, and the *Consistency* reached 100%. Though this marginal improvement in the *Accuracy* is within the uncertainty in that metric, it means that we accomplished our stated goal: to streamline the network architecture while improving or, at the minimum, maintaining performance.

The network architecture and hyperparameters established in Stage 2 were held fixed for the detailed performance evaluations in Stage 3.

### Stage 3: Assessing *ConcNet* ’s Performance

The third and final stage involved carefully assessing the performance of *ConcNet*  2, including quantifying the uncertainties associated with its performance metrics. We were specifically interested in assessing how well *ConcNet*  2 would generalize as the training, validation and testing datasets are varied. To that end, we adopted the standard statistical procedure of *Monte Carlo cross validation*^76^. The procedure we followed is described in “Methods” section. In brief, we started with an identical copy (in terms of architecture and hyperparameters) of *ConcNet*  2, the final network configuration from Stage 2. We trained the network using all the raw90-rsEEG samples from 24 participants drawn *randomly* from the 27 concussed participants, and an equal number of participants drawn randomly from the 35 control participants. The remaining participants were used for testing the network’s performance after training. The Monte Carlo procedure of generating datasets and then training and testing *ConcNet*  2 with the resulting datasets was repeated 100 times. Training on different subsets leads to variations in the values of the internal parameters of the networks. This, in turn, results in a statistical scatter in the networks’ performance during testing. We used the distributions of the various performance metrics to assess *ConcNet*  2’s performance. Specifically, we determine and report here the median values of the performance metrics as well as the 25% ($Q_{1}$) and 75% ($Q_{3}$) quartile values.

Table 4 shows the median values ($Q_{2}$), as well as the 25% ($Q_{1}$) and 75% ($Q_{3}$) quartiles, of the standard metrics commonly used to gauge a classifier’s performance. Notably, the *Accuracy* and *Specificity* remain high, with median values at 0.917 $(Q_{1}=0.833, Q_{3}= 1.000)$ and 0.900 $(Q_{1}=0.850, Q_{3}= 1.000)$ respectively. All three *Recall* quartiles are equal to 1, while the mean is also reassuringly high at 0.93.

**Table 4:** The ensemble median ($Q_{2}$), the 25% percentile ($Q_{1}$), and the 75% percentile ($Q_{3}$), of the distributions of the various network metrics. The statistics are derived from the Monte Carlo cross validation tests performed in Stage 3, quantifying the performance of *ConcNet*  2.0. The results for the five participants who were systematically misclassified have *not* been excluded.

Metric	$Q_{2}$	$Q_{1}$	$Q_{3}$	Metric	$Q_{2}$	$Q_{1}$	$Q_{3}$
*Accuracy*	0.917	0.833	1.000	*Area Under Curve (AUC)*	0.971	0.963	0.975
*Recall* (TPR)	1.000	1.000	1.000	*Informedness*	0.900	.800	1.000
*Precision* (PPV)	0.667	0.500	1.000	*Markedness*	0.667	0.500	1.000
*Specificity* (TNR)	0.900	0.850	1.000	*Miss Rate* (FNR)	0.000	0.000	0.000
*False Discovery Rate* (FDR)	0.333	0.000	0.500	*False Positive Rate* (FPR)	0.100	0.000	0.150

**Figure 4:**
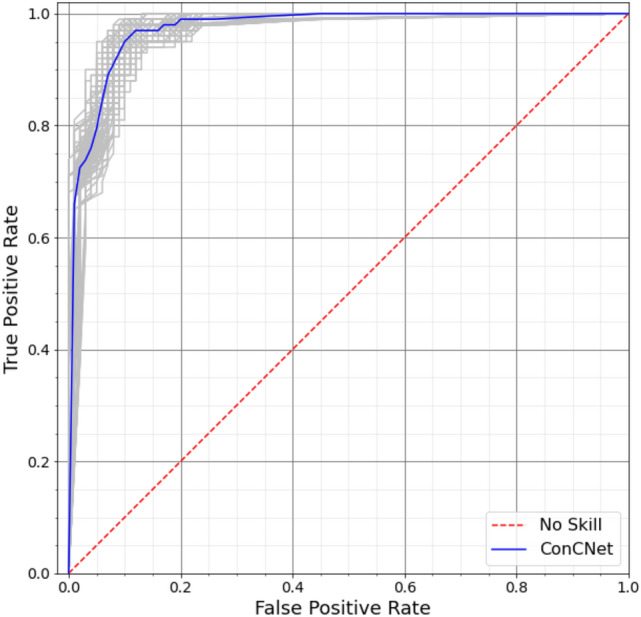
The Receiver Operating Characteristic (ROC) curves for the 100 LSTM networks. The grey curves show the results for each of the 100 networks and the blue curve shows the median. The ensemble median Area Under the Curve (AUC) is 0.971 (the 25% and the 75% percentile are 0.963 and 0.975, respectively). A classifier with no better accuracy than chance would be expected to have an AUC of 0.5, while an AUC of 1 corresponds to a classifier with perfect accuracy. A value of $>0.96$ indicates a classifier with excellent discriminatory ability^78^. (*Plotted with Matplotlib v3.2.1*^75^).

We also examined the Receiver Operating Characteristic (ROC) curve and the corresponding Area Under the Curve (AUC) for *ConcNet* 2. ROC and AUC are standard performance metrics for classifiers (see e.g.,^77^ ). They are particularly useful because they are independent of the adopted threshold for classification, in contrast with the values of *Accuracy*, *Specificity* and *Recall*. Figure 4 shows the ROC curves derived from cross validation. It shows the True Positive Rate (TPR) vs. the False Positive Rate (FPR) for a range of classification thresholds; a low classification threshold generally leads to more control participants being classified as concussed. The AUC is also calculated for each of the ROC curves. The ensemble median AUC for *ConcNet*  2 is 0.971 $(Q_{1}=0.963, Q_{3}= 0.975)$. AUC measures the performance across all classification thresholds: An AUC value of 0.5 corresponds to a classifier whose accuracy is no better than a coin toss, while an AUC of 1.0 corresponds to a perfect test (i.e. 100% recall and 100% specificity). For reference, an AUC value of $>0.96$ indicates a classifier with excellent discriminatory ability^78^.

**Figure 5:**
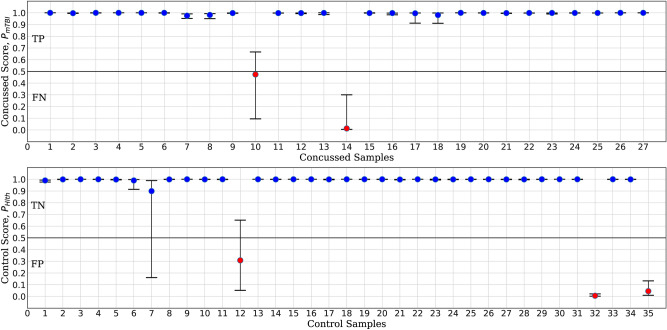
Statistics of the output layer scores for each participant in the concussed (upper panel) and control (lower panel) groups. The scores distributions are obtained from the Monte Carlo cross validation tests in Stage 3. The statistics shown include the median (circle markers) and the 25th and 75th quartiles (whiskers) of the concussion scores $P_{\mathrm{mTBI}}$ for the 27 concussed participants (upper panel). For the 35 control participants, the statistics involve the median and respective quartiles of the complementary scores $P_{{{\text{Hlth}}}} \equiv (1 - P_{{{\text{mTBI}}}} )$. Correctly classified participants (blue markers) have a median concussion score exceeding the threshold of 0.5 (concussed group) and respectively below 0.5 (control group). Correct classifications include true positives (TP) in the concussed group and true negatives (TN) in the control group. Red markers correspond to individuals who were misclassified based on their median scores; these involve two false negatives (FN) in the concussed group and three false positives (FP) in the control group. (*Plotted with Matplotlib v3.2.1*^75^).

**Figure 6:**
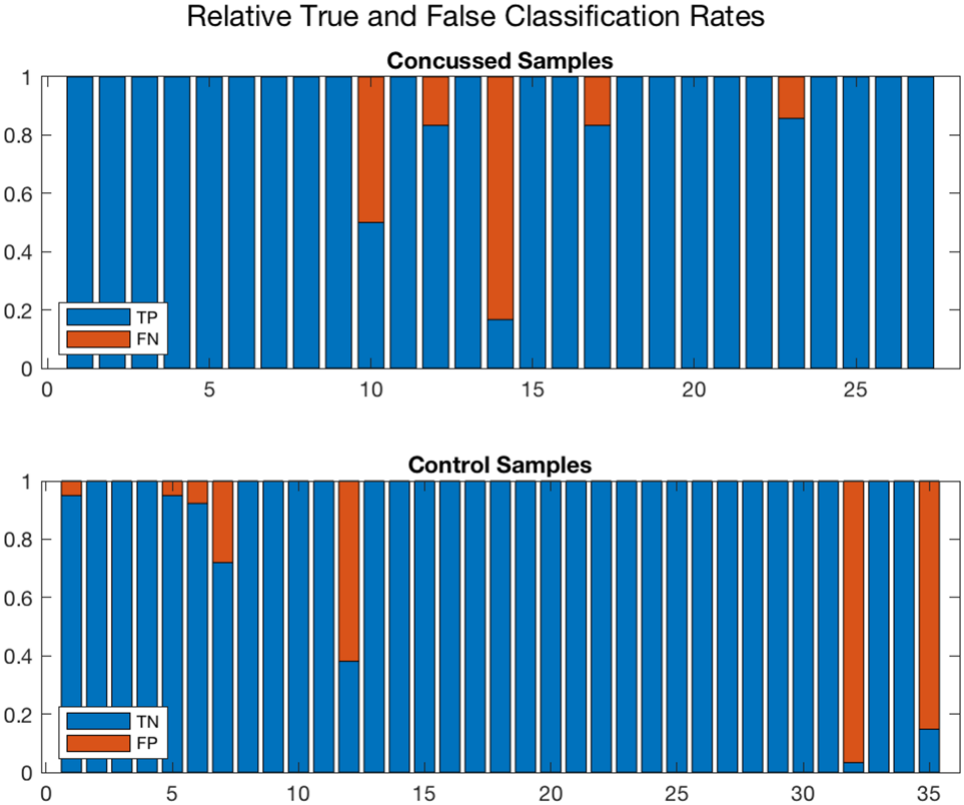
A different representation of the Monte Carlo cross validation test results for the 27 concussed and 35 control samples. The blue and red regions in each column illustrate the relative fractions of times that a participant was properly or wrongly classified by the ensemble of networks. This figure is complementary to Fig. 5 which shows the median and quartile scores for each participant. Three control and two concussed participants tended to be systematically misclassified by the networks in the ensemble. A handful of other participants were sometimes misclassified by one or two of the networks. On further examination, the training of the latter involved an EEG sample from one of the misclassified individuals. (*Plotted with Matlab R2020a*^79^).

To investigate *ConcNet*   results further, we examined the full set of predicted scores assigned to each participant. We note that a participant is assigned a score only when a raw90-rsEEG recording from that participant is presented to a network as a test case. Figure 5 shows the three quartiles, i.e, the median, the 25th and the 75th percentiles of the predicted scores distributions. For clarity, the results are divided across two panels: the top panel shows the statistics of the concussed score, $P_{\mathrm{mTBI}}$, for the 27 concussed participants, and the bottom panel shows the statistics of the control score, $P_{{{\text{Hlth}}}} \equiv 1 - P_{{{\text{mTBI}}}}$, for the 35 control participants. Blue markers represent participants who were correctly classified based on their median scores. These include *true positive* (TP) classifications in the concussed group and *true negative* (TN) classifications in the control group. Red markers represent incorrectly classified participants, i.e., two *false negative* (FN) classifications in the concussed group and three *false positive* (FP) in the control group. Note that in almost all cases of correctly classified participants their scores have very low scatter, whereas in the case of incorrect classifications three out of the five participants exhibit considerable scatter. Many of the concussed and control participants are consistently assigned a score of $P_{\mathrm{mTBI}}=1$ or $P_{\mathrm{Hlth}}=1$, respectively. Hence, their quartiles appear as merged lines. This is highlighted in the complementary Fig. 6, where the distributions of classification scores are presented as a function of the number of trials for each individual participant.

Analyzing the misclassifications further, we noted that they were *not random occurrences* in the sense that the same five individuals tended to be misclassified regardless of which of the participants’ raw90-rsEEG recordings was tested during cross-validation. To further illustrate this, we show in Fig. 6 the scores as stacked bar charts for all the concussed and control samples. For each bar, the blue and red areas represent the fraction of times that the participant under consideration was respectively correctly or incorrectly classified in the cross-validation tests. Any bar whose red portion occupies > 50% of the column corresponds to a participant who was more frequently misclassified. The two concussed and the three control misclassified participants readily stand out.

Figure 6 also shows a few additional columns with short red segments. These segments represent a small number of times that a participant was misclassified during the cross-validation tests. We found that these failures were also largely due to the misclassified samples highlighted above. In the cross-validation, since the partitioning of the full dataset into training and testing datasets is done at random, every so often the EEG data from one or more of the five consistently misclassified cases also appeared in the training subsets for some of the networks. This degraded the performance of the affected networks. The moderate value for the *Precision* and *Markedness*, and the spread in their values, are primarily due to these same five individuals. As a test, we removed the five systematically misclassified individuals from our dataset, and retrained and tested a single network. Not surprisingly, the overall accuracy rose to 98%, an improvement of over 5% from our previous median *Accuracy* of 92.6%.

Ordinarily, a small number of misclassifications are expected in any statistical machine learning model. In the present case, however, the misclassifications are not limited to a single raw90-rsEEG data sample from many different participants. Rather, the raw90-rsEEG samples from the five participants in question are consistently misclassified. This is particularly intriguing since EEG signals are well known to be strongly non-stationary on timescales greater than $\sim$0.25 s; this means that the statistical properties, including the mean and the variance of the signal in the individual samples, change with time^66,67^. Due to this non-stationarity, it is surprising to obtain consistent misclassifications from the same individuals. This result motivates a closer examination of systematically misclassified individuals to rule out potential gaps in their reported history and to build confidence for the use of *ConcNet*-like classifiers in clinical ecosystems. We will return to this matter in the Discussion.

## Discussion

The results presented here demonstrate that an LSTM-based deep learning network for concussion classification is a promising engine for an objective, brain-based platform, which can identify acute post-concussed individuals within 1 month of injury. The excellent performance of our implementation, *ConcNet*  2, serves as a proof of concept, demonstrating that a deep learning network can be trained to accurately recognize acute post-concussed individuals using only their resting state EEG data. The participants were neither required to perform a specific task nor to respond to a stimulus during data collection, and the acquired EEG data were neither filtered, cleaned of artefacts, nor subjected to explicit feature extraction. The classifier’s final network architecture is shown in Fig. 3.

*ConcNet*  2 was trained and tested using a total of 216 EEG samples, obtained from 27 male, adolescent athletes with sports related concussion within 1 month of injury, and 280 samples from 35 age-matched non-concussed (control) athletes. In our tests, the network achieved an *Accuracy* of 92.6%. Applying Monte Carlo cross validation, we carried out a detailed statistical assessment of the classifier’s performance. In these tests, *ConcNet*  2 achieved median values of 0.917, 0.900 and 1 on Accuracy, Specificity and Recall respectively (see Table 4 for more details). The magnitudes of all three are high. These metrics, however, depend on the adopted threshold for classification. Therefore, we also evaluated *ConcNet* ’s Area Under the Curve (AUC), a performance metric that is independent of the adopted threshold. The resulting ensemble-median AUC value of 0.971 confirms that the classifier accurately distinguishes between the concussed and the non-concussed participants, in the context of the population group on which it was trained.

Of the 27 concussed participants considered in this study, 25 were consistently classified correctly, while 2 participants were misclassified as control. We are intrigued by the consistent differences in the scores assigned to the raw90-rsEEG data samples from the 2 individuals in question, compared to the scores assigned to the raw90-rsEEG samples for the other 25 concussed participants. Below we discuss some possible explanations for these misclassifications.

Firstly, we cannot exclude outright the possibility that the few instances of erroneous classification are due to statistical fluctuations which affect all machine learning methods. However, the misclassifications are not randomly distributed across the participants: they are all linked to the raw90-rsEEG data samples contributed by two participants. These samples affect classification in two ways: (1) they are misclassified if they appear in the test set; (2) they reduce the classification efficacy of *ConcNet*  2 if they appear in the training set. Consequently, we consider it unlikely that the *ConcNet*  2 misclassifications are due to random errors.

Secondly, it is possible that there is a bias in the misclassified participants’ EEG dataset. One potential source of bias is a participant-specific issue with data acquisition that affects the full five minutes long EEG dataset but is unrelated to the participant’s brain state/injury status. This could happen if, for example, one or more of the sensors are not functioning properly during the data collection session giving rise to “bad channels” or the sensor cap is misaligned. At the start of our study, we did a cursory examination of all 64 power spectral density plots from each of the participants in our study to verify data quality and to identify datasets affected by “bad channels”. None of the datasets under consideration exhibited readily apparent abnormal features or anomalous trends. Nonetheless, we cannot exclude the possibility that the misclassified datasets are affected in a more subtle fashion as would happen if the cap was misaligned.

Another potential source of bias is that the two concussed participants may have been misdiagnosed at the outset. Our criteria for identifying participants with concussion were (1) documentation of the date and time of a direct blow by the team coach, followed by (2) a diagnosis by an experienced physician. While we cannot completely exclude the possibility of an error in the diagnosis, we think it is highly unlikely. However, these two participants and a third one (who, nonetheless, was consistently classified correctly by *ConcNet*  2) were scanned one month post-injury. All the other participants were scanned 1 week post injury. Since *ConcNet*  2 was trained with far fewer samples from the nearly-one-month post-injury participants compared to the bulk of one-week-post-injury participants, the network learnt to correctly classify the latter. This may be mitigated by two approaches: (1) provide more samples for the 1 month post injury group to balance the training dataset, and/or (2) maintain a strict data collection protocol, collecting data 1 week post injury only, so that the network need not learn to cope with different collection scenarios.

It is conceivable that misclassified participants who were scanned one month post-injury were either well on their way to recovery at the time of scanning or that their brains were only minimally affected by the injury. In reviewing the two misclassified concussed participants’ other available data, we found that they were indistinguishable from the other concussed participants except for the time between diagnosis and scanning. This resonates with the “on the way to recovery” conjecture. In light of this, perhaps a more pertinent question is not why the two participants were misclassified, but rather why the third participant in the 1-month post injury cohort was correctly classified. We speculate that this participant may have suffered a more pronounced injury or may have been experiencing a more prolonged recovery. Neuroimaging studies find that the brain’s response to an mTBI (as suggested by a significant spread in the measures derived from neuroimaging probes of the brain’s structure and function), varies considerably from participant to participant both in the acute phase as well as in the brain’s recovery trajectory^80,81^. This explanation may also apply to the three participants in the control group who were misclassified as concussed. Longitudinal neuroimaging studies show that in some individuals, mTBI induced changes in the brain can persist for several months and even past a year^16–24^. These persistent alterations are seen in EEG, fMRI and DTI data. We suspect that our misclassified “control” may have previously suffered one or more concussions which led to persistent changes. We did not request our control participants’ concussion history beyond two years since there is considerable evidence showing that self-reported histories are error prone^82,83^. Ultimately, the resolution of this puzzle requires analyzing a larger sample that encompasses the full range of diversity present during the acute post-concussion phase.

Another question emerging from our study is the following: what enables *ConcNet*  2 to leverage raw, highly non-stationary, EEG time series data to distinguish between concussed and control adolescent participants? The fact that the main advantage of LSTM-based networks is their ability to identify temporal correlations and causal relations within a sequential datastream suggests that *ConcNet*  2 identifies alterations in the temporal dynamics of the brain due to mTBI. Existing evidence supports the notion of mTBI-induced changes in brain dynamics: By applying the sliding window analysis to resting state fMRI data, Muller and Virji-Babul^84^ identified three distinct brain states and showed that, while non-concussed adolescents switched dynamically between the three brain states and spend approximately the same amount of time in each state, in concussed adolescents only one brain state appears most of the time. In addition, using a novel measure of non-stationarity, namely the Shannon entropy of the peak frequency shifting, Cao et al.^67^ showed that EEG non-stationarity is reduced in individuals with an mTBI. The possibility of using neural networks, like our LSTM-based *ConcNet*  2, to probe brain dynamics is an exciting new frontier that we are starting to explore.

Although promising and potentially exciting, the study described here has two main limitations that we will be addressing in future work. The first of these is that the EEG data set used to train and test the network is relatively small and was acquired exclusively from male adolescent athletes. As such, it remains to be seen whether *ConcNet* (or a variant thereof) can be successfully broadened to encompass not only female athletes but also younger and older participants. The outcomes from the current study provide a strong incentive for doing so. In fact, we were actively collecting data on female athletes before the COVID-19 pandemic forced a significant scaling back of competitive sports activities. Consequently, we decided to proceed with a male-only sample as a starting point for a proof-of-concept study demonstrating that it is possible to use brain-based signals to distinguish between concussed and non-concussed individuals. We will resume data collection (as per local public health COVID-19 protocols and guidelines) and expand our database as described.

The second limitation is that LSTM networks are very difficult to analyze, and thus it is not straightforward to identify the signatures that guide the classification. We are confident that *ConcNet* responds to changes in the brain-based EEG signals due to concussion—after all, there are clear brain-based, group-level changes between concussed and non-concussed groups: a number of quantitative variables such as graph theory measures of brain’s functional connectivity, the distribution of information flow rates in the brain, the distribution of power in the spontaneous brain oscillations across different frequency bands, etc. show significant group-level differences between the concussed and non-concussed cohorts (c.f.^25,29,34–36,50,51,67^). However, we cannot at present definitely rule out the possibility that the classification is based on (1) systematic differences in the raw EEG signals of the concussed and the control groups that do not originate in the brain but are nonetheless linked to concussion, or (2) that the EEG measurements from the concussed participants are systematically biased compared to those from control participants. Possible examples of the first include the degree of facial electromyography (EMG) contamination, frequency of eye blinks or eye movements, etc. While knowing whether the classifier reacts to differences in the electrical activity originating in the brain or other biological, albeit concussion-caused, artefacts is important from a neuroscience perspective, it is less of an issue when designing a clinical classification tool, where reliability, accuracy and consistency are much more important. With respect to systematic biases, we took a number of precautions to minimize that possibility. For instance, members of the control and the concussed groups are all athletes who play organized sports on a regular basis. Both control and concussed participants were recruited on an ongoing basis over a span of 2 years. We enforced strict adherence to the inclusion/exclusion criteria (see “Methods” section, “Participants” section), confirmed that none of the participants wore braces or permanent retainers, and ensured that the EEG data for all participants were collected in the same room/lab at the University of British Columbia using the same set of equipment and set-up. Nonetheless, it is possible that some systematic instrumental or environmental bias is contributing to the differences in the two groups’ raw EEG signals. We are undertaking a thorough analysis of the EEG data, including assessing the relative importance of each individual EEG channel. We will report on the results of this ongoing investigation in a subsequent publication.

To summarize, in this paper we introduced *ConcNet*  2, a high-performing deep learning Long Short-Term Memory (LSTM)-based recurrent neural network that is able to distinguish between non-concussed and acute post-concussed adolescent athletes using only a short (i.e. 90 seconds long) sample of resting state, raw EEG data as input. This is the first instance of a high-performing, objective, brain-based platform for automatic classification of concussion at an individual level that relies only on easy-to-acquire raw, resting state EEG data. It represents a highly promising first step towards addressing a fundamental challenge in concussion diagnosis: the dependence on highly subjective self-reporting of symptoms when determining whether an athlete has experienced a concussion.

## Methods

### Participants

The EEG recordings used in this study were collected from a total of 62 male adolescent athletes. Of these, 35 were from a non-concussed, age-matched cohort of athletes [mean age: 14.7 years; standard deviation (SD): ±2.1 years]. The other 27 individuals [mean age = 13.5 years; (SD): ±2.6 years] were athletes who had suffered a sport-related concussion, had undergone a clinical assessment by the team physician or a physician with expertise in concussion within a week of injury, and were found to meet the concussion diagnostic criteria consistent with the Berlin consensus statement^13^. The latter includes: (1) Documentation of the date and time of the injury by the team coach. Here, injury refers to either a direct blow to the head, face, or neck, or a direct blow to the body that leads to an impulsive force transmitted to the head, resulting in changes in one or more of the following clinical domains: (1) physiological (e.g. neck pain, balance problems, headache, fatigue), (2) cognitive (e.g. difficulty with attention, feeling in a “fog”), (3) emotional (e.g. irritability, sadness, depression ) and (4) behavioural (i.e. sleep/wake disturbances). (2) An assessment at the time of diagnosis of the injured athlete using either the Child Sports Concussion Assessment Tool 3 (Child SCAT3), if the injured athlete was younger than 13 years of age (https://bjsm.bmj.com/content/bjsports/47/5/263.full.pdf), or using the Sports Concussion Assessment Tool 3 (SCAT3), if 13 years or older (https://bjsm.bmj.com/content/bjsports/47/5/259.full.pdf). Both SCAT3 and Child SCAT3 are standardized concussion and concussion symptom assessment tools, which combine self-reporting of the number of symptoms experienced and their severity with cognitive, behavioural, physiological and emotional assessments based on a combination of clinically-accepted objective diagnostic tools and self-reporting of symptoms. Finally, the Berlin consensus statement requires (3) a clinical examination as well as a review of all the available information, by an experienced physician.

EEG data were acquired from the concussed athletes within a month of injury. We chose the time window of one month based on the 2017 Berlin Consensus Statement’s expected time for clinical recovery after injury in children and adolescents^85^. All of the injured participants were re-assessed using the SCAT3/ChildSCAT3 at the time of data collection, and we acquired data only from those who were symptomatic. Individuals with focal neurologic deficits, pathology and/or those on prescription medications for neurological or psychiatric conditions were excluded from this study. Additionally, we also excluded all participants who had braces or permanent retainers.

The data collection for both the control and concussed groups occurred over a span of 2 years. The recruitment for the study was ongoing during this time frame and participants were invited to participate anytime during this period as long as they met the inclusion/exclusion criteria. EEG data for all participants were collected in the same room/lab at the University of British Columbia using the same equipment and set-up.

Our study was approved by the University of British Columbia Clinical Research Ethics Board (Approval number: H17-02973). All participants provided assent and the adolescents’ parents gave written informed consent for their children’s participation under the approval of the ethics committee of the University of British Columbia and in accordance with the Helsinki declaration.

### An overview of the analysis pipeline

We first briefly summarize the steps that we followed for (1) data collection and segmentation as well as (2) network training, validating and testing. In the subsequent subsections, we provide more details about each step and we explain our specific choices in cases where more than one algorithm or approach were available.

1. Collected five minutes of resting state EEG data from each participant under eyes closed condition using a 64-channel EEG geodesic cap and a high-impedance amplifier. Data acquisition was started only after all the scalp electrode impedances were confirmed to be below 50 $k\Omega$,
2. Trimmed 4 s of data from the start and end of each time series (total = 8 s). We did not otherwise process the EEG time series data; i.e. we neither filtered nor cleaned the data to remove artefacts due to line noise, eye blinks and motion, and electromyogram (EMG) contamination.
3. Extracted eight 90 seconds long sets of synchronous segments from each of the 64-channel time series comprising each individual’s raw resting state EEG dataset.
4. Identified the LSTM-based deep learning network as the most promising architecture and settled on the basic network design shown in Fig. 1.
5. Selected an initial “egalitarian” classification threshold of 0.5 so that input data with classification scores $\ge 0.5$ are labeled as concussed while those with scores $< 0.5$ are classified as control.
  *STAGE 1: Initial Training and Preliminary Assessment of the LSTM Network*
6. Generated a randomly selected subset of 19 (of 27) concussed and 25 (of 35) control participants.
7. Divided the subset of concussed participants into a random 80:10:10 (i.e., 15:2:2 participants) training-validation-test split. Each split included all eight raw90-rsEEG segments from the associated participants.
8. Randomly divided the subset of 25 control participants (and their associated eight raw90-rsEEG segments) into training-validation-test groups (i.e., 15:2:8 participants split) to maintain an equal number of control and concussed participants in the training and validation sets.
9. Used the standard *mini-batch stochastic gradient descent* (SGD) method to train the network, focusing exclusively on the network’s internal weights, biases and learning rate during the current stage. Over the course of training, the network parameters were updated using *Adam*, an adaptive optimization algorithm.
10. Tracked the evolution of the network’s error function over the course of training using the validation dataset. Once the error function dropped below a preset threshold ( $< 10^{-4}$) and remained stable, training was concluded.
11. Assessed the “trained” network using the testing dataset, followed by a second test using 27 raw90-rsEEG samples drawn randomly from the 8 concussed and 10 control participants, whose data were not used during the above training-validation-test cycle. The classifier was considered as acceptable if its accuracy exceeded the threshold of 80%.
  *STAGE 2: Optimizing Network Hyperparameters*
12. Used direct grid search to tune the network’s principal hyperparameters. These include the number of LSTM layers, the number of bi-LSTM units per layer, the number of Fully Connected layers and the number of nodes in each, the number of Dropout layers and the associated dropout fraction, and the learning rate.
13. For each set of hyperparameters, the same training and validation procedure as in Stage 1 was carried out with one difference: all of the available data were used to construct the training-validation-test datasets. The training set comprised all eight raw90-rsEEG segments from 21 randomly selected concussed and 21 randomly selected control participants; the validation set consisted of all eight raw90-rsEEG from 3 concussed and 3 control participants randomly selected from the balance of the participants; and the remaining participants (and their segments) made up the testing set. The optimal set of hyperparameters was determined by maximizing the network’s classification accuracy. The architecture of the optimized (final) network, *ConcNet*, is shown in Fig. 3. *STAGE 3: *
  *Evaluating Network Performance Using Monte Carlo Cross Validation*
14. Generated an ensemble of 100 identical clones of *ConcNet*.
15. Trained and tested each network using a random 90:10 partition of the full dataset, subject to the constraint that there are an equal number of concussed and control participants in the training dataset.
16. Maintained a detailed log of which concussed and control participants were used to train and test each of the networks; each individual network’s performance metrics; and how each participant was classified by the different networks in the ensemble during the testing phase.

### EEG recordings

#### Data collection

Five minutes of resting state EEG data were collected while participants had their eyes closed. We used a 64-channel HydroCel Geodesic Sensor Net (EGI, Eugene, OR) connected to a Net Amps 300 high-impedance amplifier with a $0.1\;\mathrm{Hz }$ high-pass analog (i.e., hardware) filter. The signals were referenced to the vertex (Cz) and recorded at a sampling rate of 250 Hz; also, data acquisition was started only after all the scalp electrode impedances were confirmed to be below 50 $k\Omega$^86^, in keeping with the recommendations for the EGI Net Amps 300 high-impedance amplifier^87^. To eliminate any transients at the start and the end of a data collection session (due, for example, to participants taking a few seconds to settle down, etc.), 1000 data points were removed from the beginning and the end of each time series; this corresponds to removing data with a total duration of 8 seconds. We did not further filter or otherwise clean the data to remove artefacts due to line noise, eye blinks and motion, and electromyogram (EMG) contamination as is typically done (c.f. Porter et al.^88^, Rotem-Kohavi et al.^52,53^, Munia et al.^20^). The resting state EEG data were acquired in binary simple (.*RAW*) format^89^, and converted to *Matlab*^79^ (.*mat*) format using *EEGLab*^90^ for further processing and analysis.

#### Data segmentation and augmentation

As a first step, we segmented the 64-channel EEG data into three, 90-s long consecutive sets of raw90-rsEEG. As noted previously, EEG signals exhibit substantial non-stationary behaviour in both the temporal and the spectral domains^65–67^. Normally, this behaviour poses a significant challenge for extracting stable features from the signal.

In the present study, however, we leveraged non-stationarity to augment our data compilation by extracting, from each participant’s five-minute long 64-channel data stream, five additional 90-second long data segments with randomly chosen starting points. In total, we extracted 8 sets of raw90-rsEEG recordings per participant, with each set comprising synchronous segments from each of the 64 time series constituting each individual’s raw resting state EEG dataset. Henceforth, we refer to each raw90-rsEEG data set as a *sample*. The conversion and the extraction of the eight raw90-rsEEG were done using *Matlab*^79^ scripts. These scripts can be found at https://github.com/Karuntg/RNN4ConcClass.git.

Our choice to use 90-second long segments reflects a compromise between wanting to use a short EEG sample on the one hand, and on other hand, ensuring that the sample is sufficiently long to capture low frequency brain activity, i.e. oscillations with frequencies down to 0.1 Hz. The latter constraint was motivated by two considerations: (1) EEG studies find that, compared to non-concussed controls, concussed athletes exhibit altered activity in the delta band (0.5–4 Hz)^20,34,91^, with increasing divergences observed near the lowest frequencies. (2) Among adolescents, one of the resting state networks strongly impacted by concussion is the default mode network^21,51,92^. This network has been shown to support low frequency, large amplitude oscillations^93^.

### Behavioural data

As noted, when the EEG data were collected, the number of symptoms and symptom severity of each of the concussed participants were also evaluated using either the SCAT3 or the ChildSCAT3 concussion assessment tool. SCAT3 lists 22 symptoms that can be scored from 0 (none) to 6 (severe) while Child SCAT3 lists 20 symptoms with scores ranging from 0 (none) to 3 (often). The overall symptom severity score of the injured athletes is the sum of the individual symptom ratings. SCAT3 allows for a maximum score of 132 while the highest possible Child SCAT3 score is 60. These data were not used for any other purpose other than to confirm that the concussed athletes were symptomatic at the time of data collection.

### Network architecture

The *ConcNet*  2 classifier proposed herein uses standard network architecture as well as training, validation and testing protocols. Therefore, the following description is concise. The interested reader can find more details in, for example^74^.

As noted in “Stage 1: Identifying a promising machine learning algorithm for *ConcNet*” Section, we had previously experimented with several popular machine learning classification algorithms, including the support vector machine (SVM) algorithm^43^ and the simplest type of multilayer perceptron (MLP) neural network^47^. The latter was a three-layer network consisting of an input layer that receives the data, a single hidden layer that processes the data, and an output layer that classifies based on the results from the hidden layer. The input for both algorithms consisted of a set of statistical measures (hereafter, *features*) extracted from each participant’s 64-channel resting state EEG time series data. Neither of these two algorithms yielded classification accuracy above 65%. This is not completely surprising due to the non-stationarity of the EEG signals and the wide variation of EEG features between individuals belonging in the same group. It is well known that measures extracted from different segments of time duration $\Delta t$, even from a single non-stationary EEG time series collected at one sitting, can vary significantly^65–67,94^. However, prior to extracting the features, we had filtered the data^52,53,88^, an approach that is commonly adopted to mitigate weak non-stationary behaviour, removed various artefacts including line noise, etc. This works well enough when one is interested in *population-level* differences between concussed and control groups^25,29,50,51^. However, the distribution functions of features for the control and concussed individuals overlap, which hampers attempts to use the features to classify *individual* participants. The problem is further compounded by the fact that EEG time series are strongly non-stationary and simple procedures, like filtering, do not completely remove the non-stationarity. This behaviour complicates the extraction of stable features from the signal and poses a significant challenge to the design of reliable brain-computer interface systems able to operate in real environments^94–97^. In light of this conundrum, and in the interest of keeping the data processing pipeline as simple as possible, we opted to use the observed (raw) EEG signals and rely on a deep neural network to learn differences in the signals.

As for the choice of the deep learning network architecture, we took our cue from the brain-computer interface (BCI) literature and focused our attention on a particular class of recurrent neural networks, referred to as Long Short -Term Memory (LSTM) networks^68,69^, that are able to learn long-term dependencies inherent in input data sequences, such as a set of EEG time series. In the context of EEG-based BCIs, LSTM networks have been found to better capture the non-stationary behaviour of EEG time series, and they consequently yield higher accuracy in classification tasks^97^. The key feature is the inclusion of *memory units*. As a temporal sequence is processed, the memory of the results associated with prior time points persists in these units and informs the processing of current and future inputs. This ability to capture long-term dependencies is the reason why LSTM networks can successfully learn time-series data which involve complex nonlinear dynamics.

In keeping with the common Machine Learning practice of *transfer learning*, we adopted the LSTM network used in a sequence classification application^98^ as our starting point. Transfer learning refers to the practice of taking a network architecture that has been developed and successfully tested in one application, and adapting it for another similar application. In the present instance, only the final layers of the deep learning network, which directly govern the final classification output, were modified. The preceding layers which perform information preprocessing and synthesis were largely unaltered.

The main elements of our chosen network design, schematically shown in Fig. 1, consist of a sequential input layer that receives 64-channel EEG data and feeds them to two recurrent layers, each comprising 100 bi-directional Long Short Term Memory (bi-LSTM) units. These bi-directional memory units^99^ integrate information from both past and future time steps to compute the activation signals during the forward pass, as well as the updates that are applied to the network’s internal parameters (i.e., the weights and biases) during back propagation. We chose to use bi-LSTM units because bi-directionality enhances network stability, speeds up training, and leads to improved performance in comparison to networks with unidirectional computation flow^100^. Stability is especially important when training with small datasets, as in our case. To further synthesize the information output of the bi-LSTM layers, we follow standard practice in classifier design and employ Fully Connected (FC) deeper layers. Each FC node computes the weighted sum of all the outputs from the preceding layer and adds a bias. In all except the last FC layer, this weighted sum is fed to a *Rectified Linear Unit* (ReLU) activation function, and the resulting output is passed to the next layer. We have opted to use *ReLU* (Rectified Linear Unit) activation function for the FC layers because it has a wider dynamic range than other commonly used activation functions and therefore enables efficient learning in deep learning environments. The non-linear character of the activation function facilitates the learning of complex dependencies within the data.

Following each bi-LSTM and all FC layers except the last one, we applied the commonly used *Dropout* regularization technique to avoid network overfitting^101^. The Dropout layer discards a user-chosen fraction of randomly selected inputs (hereafter, the dropout fraction). The dropout fraction for all the Dropout layers was set to 30%. Finally, the weighted sums from the nodes of the third FC layer are fed to the output layer. The latter computes a linear combination of the inputs and applies a logistic function which assigns a *score* ($0 \le P_{\mathrm{mTBI}} \le 1$) corresponding to the raw90-rsEEG dataset inputted to *ConcNet*. Depending on whether the score is greater or less than a user-chosen threshold, each sample is assigned a class. We set the threshold at 0.5, so that samples with score $\ge$ 0.5 are classified as concussed (class $=$ 1), while samples with scores < 0.5 are classified as control (class $=$ 0).

### Network training, validation and testing

#### Stage 1

We used a subset of the available EEG data, i.e., all raw90-rsEEG samples from 25 (of 35) control and 19 (of 27) concussed participants, to *train* the LSTM network (i.e., determine its internal weights and biases). We divided these data into training, validation and test datasets, as follows: The set of 19 concussed participants was randomly split using a 80:10:10 prescription, resulting in 15 concussed participants (and all their associated raw90-rsEEG samples) in the training dataset and 2 participants (and all their associated samples) in each of the validation and testing datasets. We augmented the training and validation datasets with data from 15 and 2, respectively, randomly selected control participants to ensure that the two classes were balanced. (Balancing is important to mitigate potential biases arising from classes of unequal size.) The data from the remaining 8 control participants were added to the test dataset. Finally, the data from the 8 concussed and 10 control participants not included in the above partitions were reserved for further testing the resultant classifier.

To train the network, we chose to use the standard *mini-batch stochastic gradient descent* protocol (e.g., Chap. 5 of^74^). A brief description of the procedure is as follows: The training dataset, comprising of a total of 240 raw90-rsEEG data samples, is divided into 12 *mini-batches* each consisting of 20 randomly selected data samples. The internal parameters of the network are initialized to small random values. For each input training sample, the network predicts a class label during the *forward pass* based on the current values of the weights and biases. The difference between the known class of the sample and the LSTM network’s prediction is incorporated in an *error function*. During the *back propagation* step, the gradient of the error function (i.e., the vector of its partial derivatives with respect to each of the network’s parameters) is computed and used to update the parameters. The goal is to find the global minimum of the error function (and to identify the respective parameter values) by iterative optimal descent along the steepest gradient. The network parameters are updated after processing each mini-batch. For the updating we used *Adam*^102^, an adaptive learning rate optimization algorithm that performs well even with non-stationary, noisy, and small datasets.

Our decision to use the *mini-batch stochastic gradient descent* approach was guided by the fact that the conventional “gradient descent” strategy is prone to getting stuck in local minima. The *stochastic gradient descent* (SGD) can overcome this problem. There are two versions of the SGD algorithm, the standard and the *mini-batch*. In the standard SGD, one randomly drawn sample from the training set is used to numerically approximate the gradient. In the *mini-batch* SGD approach, the gradient is computed using an average over a small set of training samples in a mini-batch. This approach retains all the advantages of SGD and has been shown to exhibit more stable convergence and better generalization performance across a wide range of experiments^103^.

Following the updating of the parameters after each mini-batch is processed, we compute and record the error functions for both the training and the validation datasets, and repeat the regime until all the mini-batches have been processed. This cycle is referred to as an *Epoch*. After each epoch, the training set is shuffled and divided into new mini-batches for the next training epoch. The process is repeated for 20 epochs. Thereafter, we review the changes in the training and validation errors over the 20 epochs. We consider the training to be successful if the two error functions decrease monotonically, both drop below a user defined threshold (i.e. $< 10^{-4}$ in our case), and remain consistently below the threshold for the balance of the 20 epochs. If either error function has not decreased below the threshold or does not remain stably below threshold, the training is deemed unsuccessful. In this case, the network hyperparameters are modified and the training cycle is restarted. During the present exploratory phase, in order to assess whether the LSTM-based network was promising we only performed cursory tuning of the initial learning rate; this user-supplied initial condition for the Adam optimizer plays a key role in determining the speed of network training. More systematic tuning of all the hyperparameters was conducted in Stage 2 as explained below. Once a network has been successfully trained, we test the performance of the network with the test dataset. We designated a classifier as promising if its *Accuracy* exceeded 80%.

We note that above criterion, “*Accuracy* >80%”, was only used to decide whether the LSTM-based classifier warranted further investigation; it does not have any other function. It has no impact on the *actual* performance of the algorithm following a systematic training, validation and testing regime involving the full dataset. The adopted value of $80\%$ is *ad hoc* although it was motivated by two considerations: (1) a classifier tool must attain high accuracy in order to be clinically acceptable and $80\%$ seemed like a promising starting point; and (2) its initial performance (in terms of accuracy) was comparable to that of the convolutional neural network-based approach of Boshra et al.^48^, which uses single-trial EEG/ERP measurements to identify signs of concussion. The latter attained an *Accuracy* of $85\%$.

After identifying a promising classifier, which we dubbed *ConcNet* 1, we subjected it to a second testing regime. For this second testing, we used 27 raw90-rsEEG samples drawn randomly from the 8 concussed and 10 control participants whose data had been reserved for this purpose. Note that *ConcNet* was developed without using any data from these participants. This second test dataset was also larger and more diverse than the first test dataset, and its class labels were kept masked until after the network had processed (and classified) the data. We re-computed the network *Accuracy* based on the resulting classification results. As mentioned in “Results” section, with the bi-LSTM design, even with minimally tuning just the learning rate, *ConcNet* achieved an *Accuracy* of $88.9\%$. Full results for the present stage are discussed in “Stage 1: Identifying a promising machine learning algorithm for *ConcNet*” Section.

#### Stage 2

During this stage, our aim was to optimise *ConcNet* ’s hyperparameters and simplify the network architecture, while improving or at the minimum, maintaining its performance. The hyperparameters include the number of LSTM layers, the number of bi-LSTM units per layer, the number of fully connected layers and the number of nodes in each, the regularization scheme and dropout rate used, and the learning rate. Even though there exist automated methods for tuning hyperparameters, we opted for a direct grid search scheme. All of the data were used to construct three groups: a training data set comprising the eight raw90-rsEEG segments from each of 21 randomly selected concussed and 21 randomly selected control participants, for a total of 336 samples; a validation set consisting of the eight raw90-rsEEG from each of the 3 concussed and 3 control participants, again randomly selected from the balance of the participants (48 samples in total); and a testing set made up of the rest. As in Stage 1, this partitioning corresponds roughly to an 80:10:10 split for the concussed dataset and balances the number of concussed and control participants in the training and validation sets. The training and validation procedures are as described in Stage 1. At the end of each successful training cycle, the performance of the network was assessed using the test dataset. This was repeated for all the hyperparameter values on the search grid. The optimal set of hyperparameters was determined by maximizing the network’s classification accuracy. The hyperparameter tuning exercise resulted in a marginal improvement in the performance metrics, with the *Accuracy* increasing to 92.6%. However, it is important to point out that the improvement was achieved with a simpler, more streamlined network architecture. The optimal design (see Fig. 3) involves fewer FC layers and fewer nodes per FC layer than the architecture tested in Stage 1; in addition, the FC layers of the optimal design are not followed by Dropout layers.

#### Stage 3

In this stage, we used *Monte Carlo cross validation* to assess how *ConcNet* ’s performance would generalize to more heterogenous datasets, as well as to quantify the uncertainties in the performance metrics. Specifically, we made 100 clones of the final *ConcNet*  2 configuration. All 100 networks employed the same architecture and hyperparameters established in Stage 2. Each network was trained and tested on a random 90:10 split of the total available data, ensuring to maintain a balance between the numbers of concussed and control participants in the training dataset; since we were not tuning the hyperparameters, there was no need for a validation dataset. The training and testing procedures were identical to those used in Stage1/Stage 2. Due to the randomness of the training dataset fed to each network, the estimated internal weights and biases are expected to vary, as will the individual network performance during testing. This testing design yields a statistical distribution for each performance metric, from which we calculate the median value and the associated uncertainty (based on the 25th and 75th quartiles).

During Stage 3, we maintained a detailed log of (1) which concussed and control participants were used to train and test each network, (2) the classification scores for each test sample output from the final layer during testing, and (3) the predicted labels. We also calculated the performance metrics for each of the networks. This type of assessment is critical for determining how well *ConcNet*  2 would generalize to larger, more heterogeneous datasets.

*ConcNet* was implemented using the Deep Learning Toolbox^104^ available in *Matlab* R2020a^79^ (see also^98,105^). Network training and testing as well as the analysis of the results were implemented using scripts which can be downloaded from https://github.com/Karuntg/RNN4ConcClass.git. Contact the corresponding author for access.

## Supplementary Information

Supplementary Information.
