# Supplementary material for: Recurrent neural network-based acute concussion classifier using raw resting state EEG data
Source: Sci Rep. 2021 Jun 11;11:12353. doi: 10.1038/s41598-021-91614-4 (PMC8196170; doi:10.1038/s41598-021-91614-4)
Supplement: Supplementary file 1 — Supplementary Information. [file 41598_2021_91614_MOESM1_ESM.pdf]

Supplementary Information
Recurrent Neural Network-based Acute Concussion Classifier using Raw Resting State EEG Data

Karun Thanjavur[1,*], Arif Babul[1,+], Brandon Foran[2+], Maya Bielecki[2+], Adam Gilchrist[2+], Dionissios T. Hristopulos[3,+], Leyla R. Brucar[4,+], and Naznin Virji-Babul[4,5,+]

[1]Department of Physics and Astronomy, University of Victoria, Victoria, British Columbia, V8P 5C2, Canada
[2]Department of Computer Science, Middlesex College, Western University, London, Ontario, N6A 5B7 Canada
[3]School of Electrical and Computer Engineering, Technical University of Crete, Chania 73100, Greece
[4]Djavad Mowafaghian Centre for Brain Health, University of British Columbia, Vancouver, British Columbia, V6T 1Z3, Canada
[5]Department of Physical Therapy, Faculty of Medicine, University of British Columbia, Vancouver, British Columbia, V6T 1Z3, Canada
[*]karun@uvic.ca
[+]these authors contributed equally to this work

ABSTRACT

This supplement contains additional methodological details pertaining to the analysis of EEG data and power spectral plots of both the control and concussed groups.

EEG Electrode Clusters

Figure S1 displays the electrode map for the EGI 64 electrode HydroCel Geodesic Sensor Net cap used in this study. The top and bottom of the array correspond to the front and the back of the head, respectively. The ovals (outlined in red) show the six electrode clusters that we used for power spectra analysis described in this Supplementary section. The clusters correspond to the following general regions of the scalp: frontal cluster encompassing the left, middle and right frontal areas of the scalp; the central left and central right clusters, the left and right temporal-parietal clusters, and the occipital cluster encompassing the left, middle and right occipital areas. Using this clustering we can show representative power spectral densities (PSDs) over the entire scalp with a reduced set of spectral plots. The individual channels associated with each cluster are listed in Table S1.

Region	EEG sensor numbers
Frontal (F)	2, 3, 5, 59, 60, 6, 8, 9, 10, 11, 12, 13
Central, left (CL)	15, 16, 20, 21, 22
Central, right (CR)	41, 49, 50, 51, 53
Temporal-Parietal, left (TPL)	24, 25, 27, 30
Temporal-Parietal, right (TPR)	44, 45, 48, 52
Occipital (O)	35, 37, 39

Table S1. The EEG sensor numbers corresponding to the six regions of the scalp.

Figure S1. Sensor layout for EGI 64 electrode HydroCel Geodesic Sensor Net. The red ovals show the six electrode clusters used for power spectra analysis. The sensor numbers are listed in Table S1. (*The electrode map is used with permission from Magstim EGI, MINNEAPOLIS, MN, and Eugene, OR, USA*).

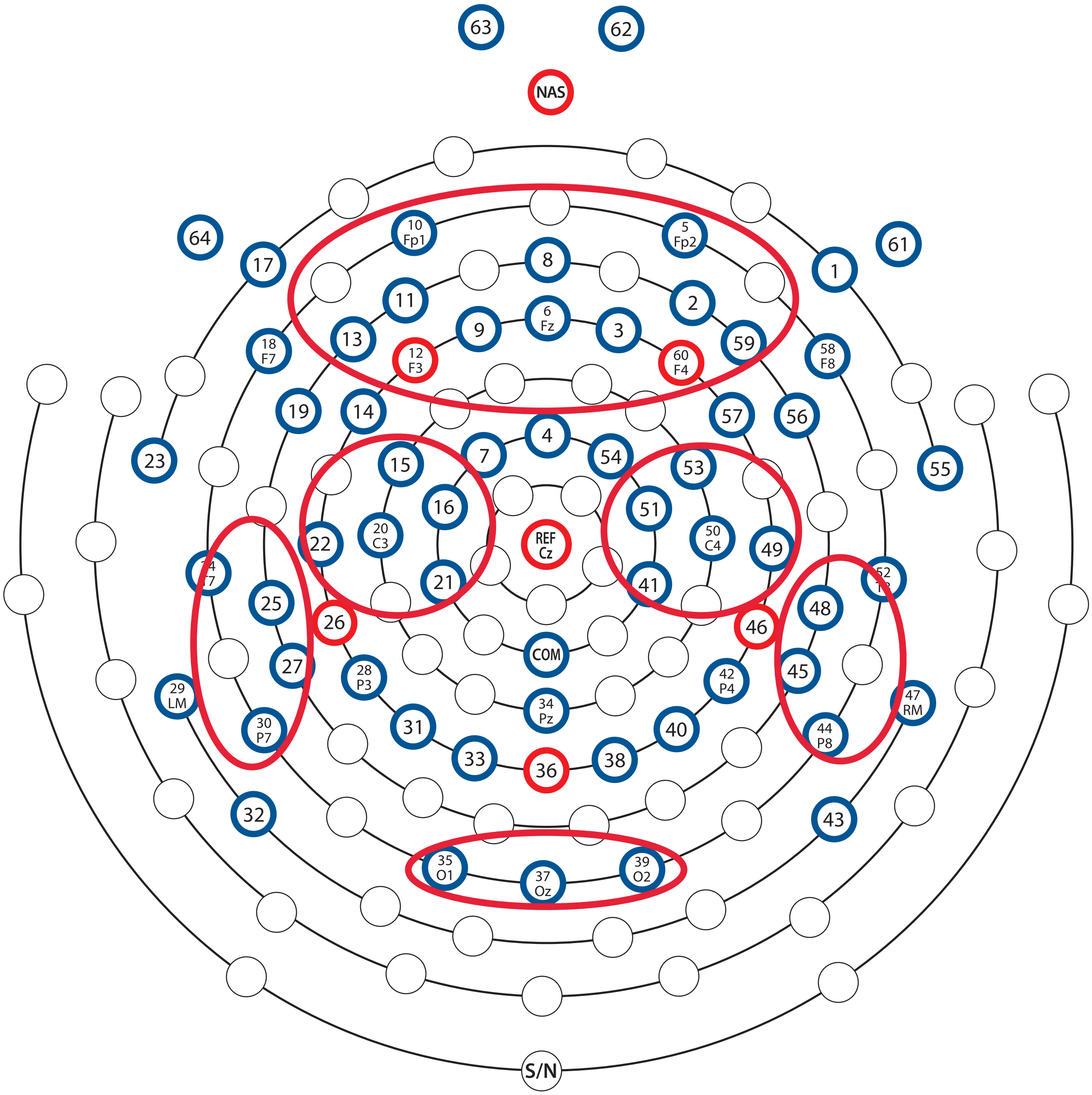

Power Spectrum Comparisons

We computed the power spectral density (PSD) of each participant's 64 *raw* EEG time series using *Matlab* [1] function, *pwelch*, and determined the median PSD for each of the six electrode clusters. The results, for the frequency range [0.1 Hz, 100 Hz], are shown as light orange (concussed) and light blue (control) curves in respective panels in Figure S2. Each row of the matrix plots shows spectral densities for the same cluster, with the PSDs of the concussed participants depicted in the left panel and the PSDs for the controls in the right panel. We also show the median PSD evaluated over all the participants for each class

(control vs. concussed) and electrode cluster. These are also plotted in each panel (in red for the concussed and in dark blue for the control participants).

A visual inspection indicates that the PSDs for the concussed and the control participants are on the whole similar. A detailed row-by-row examination of the plots reveals small differences between the median curves at specific frequencies or in specific frequency bands. Here, we will consider the three most readily apparent of these differences:

1. *Alpha Peak Frequency* (APF): APF is the frequency of a local PSD maximum in the alpha band [8 − 13 Hz]. This peak is thought to be a correlate of cognitive function, and a shift towards lower frequencies has been associated with mild cognitive impairment. In the context of mTBI, empirical results are mixed. Balkan et al. [2] did not find any APF differences between concussed and control participants. On the other hand, Dunkley et al. [3] found a trend towards lower peak frequency in the mTBI group but noted that the differences were not significant, even at the group-level. Our results are similar. We find that the APF of the median PSD of the concussed group, across all six electrode clusters, is lower than that of the control group. However, the distributions of APFs exhibit significant overlap. Considering the frontal region as an example, we note that the APFs of 9 correctly classified controls (i.e. 26% of the sample) are lower than the median APF of the concussed group.
2. *Power in the high-gamma band* : The amplitude of the median PSD of the concussed group has a slightly lower amplitude over the frequency range [65 − 90 Hz]. The shift is most apparent in the two temporal-parietal clusters and the occipital cluster. Gamma band (30 − 100 Hz) neuronal activity has been associated with a number of cognitive and executive functions. There is some evidence that gamma band activity may be altered by mTBI [4,5]. On the other hand, it is also well known that gamma band can be strongly contaminated by electromyogenic (EMG) activity of the scalp, face and neck muscles [6]. These muscles are very sensitive to emotional state and stress levels, and can experience prolonged contraction throughout an EEG recording that manifests as elevated PSD across the gamma band (see, for example, Figure 1b of Shackman et al. [7]). The temporal and frontal clusters are especially susceptible to EMG contamination. We are presently unable to argue in favor of one or the other origin (i.e., mTBI-based or EMG-based) for the differences between the two groups. We do, however, find that the distributions of broadband gamma power for both the concussed and control groups are wide and exhibit significant overlap; this is the case even for the two temporal-parietal clusters which exhibit the most pronounced differences in the amplitude of the median gamma band PSDs between the two groups. For example, seven of the correctly classified concussed participants have broadband gamma power greater than the control group's median value.
3. *70 Hz peak*: The amplitude of the 70 Hz peak (with respect to the local neighbourhood) of the median PSDs differs between the concussed and the control samples. However, as with the two metrics discussed above, the peak amplitude distributions are also broad and overlap considerably. For the temporal-parietal right cluster, for example, seven correctly classified concussed participants have 70 Hz peak amplitude that exceeds the control median value.

The fact that there are differences in median EEG power spectral density of the concussed and the control groups is not surprising. As noted in the Introduction of the main paper, there is considerable literature reporting on group-level differences (see references in main paper). However, given the significant scatter in the PSDs within each class, nobody has yet been able to use these differences, singly or collectively, to statistically differentiate between non-concussed and concussed states at the *individual* level. Even attempts to apply *Machine Learning* algorithms (e.g. Support Vector Machine) to a collection of summary spectral features extracted from EEG datasets have not succeeded in identifying concussed individuals with a high degree of confidence. In fact, it is possible that the differences between the two groups are not solely determined from two-point statistical features (such as those embodied in the power spectral density) but rather from differences in higher-order correlations.

In summary, the power spectral densities of the concussed and control participants are comparable and exhibit similar spectral features. Even though small differences in the median curves at certain frequencies in each region may be seen, there is also significant scatter clearly evident amongst the participants in each class. Using only the differences in these few features would consequently lead to significant scatter in the predicted classification scores, and thus in the performance of the network. Since *ConcNet* achieves an accuracy of >90%, it may be inferred that the classifier does not merely latch on one or more of the differences in the features, but actually uses the information in the full rs-EEG dataset of each participant.

Figure S2. Power Spectral Density of Raw EEG Data from the Six Scalp Clusters (continued on next page) (*Plotted with Matplotlib v3.2.1* [8]).

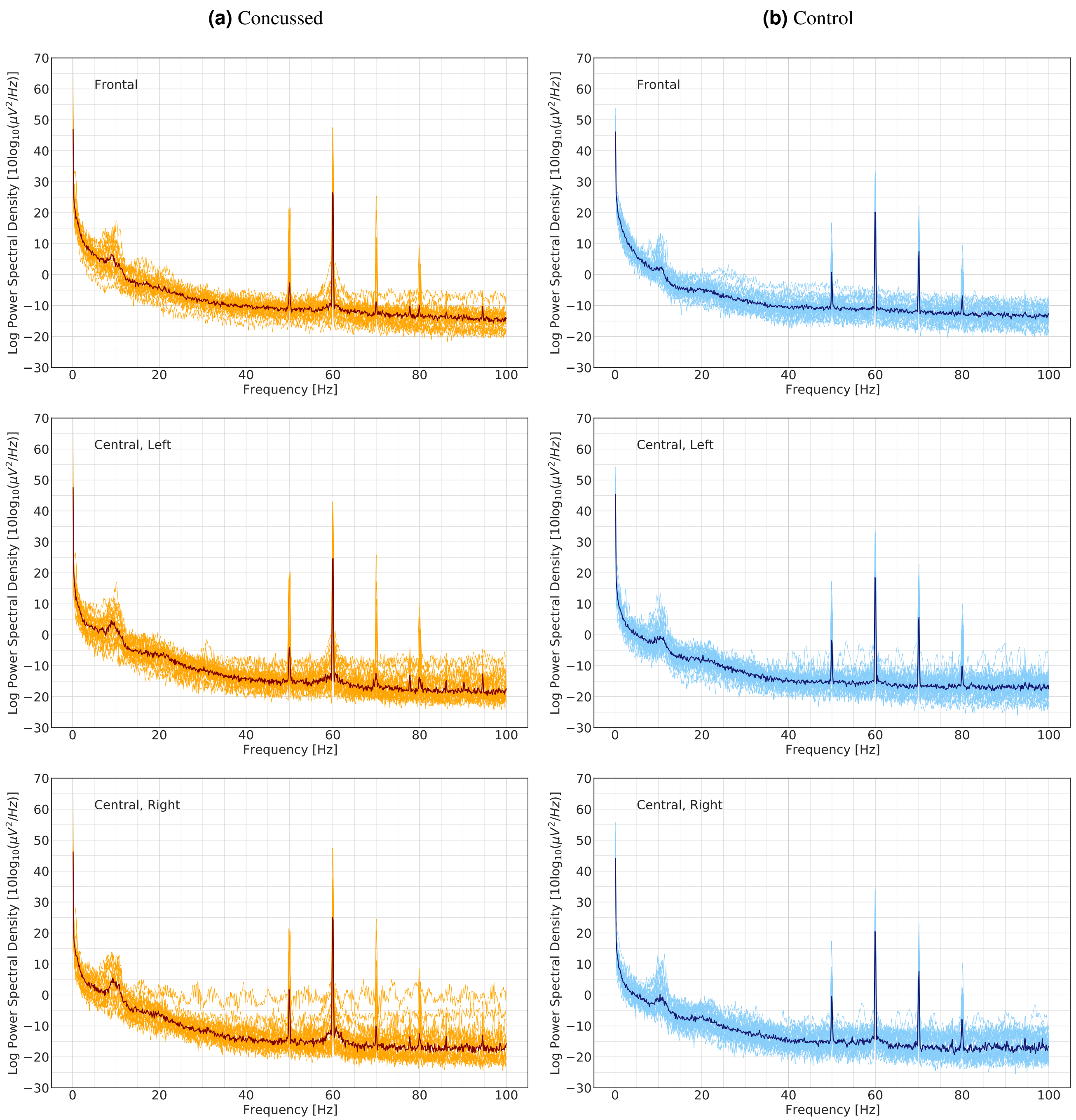

Figure S2. Power Spectral Density of Raw EEG Data from the Six Scalp Clusters (continued from previous page) (*Plotted with Matplotlib v3.2.1* [8]).

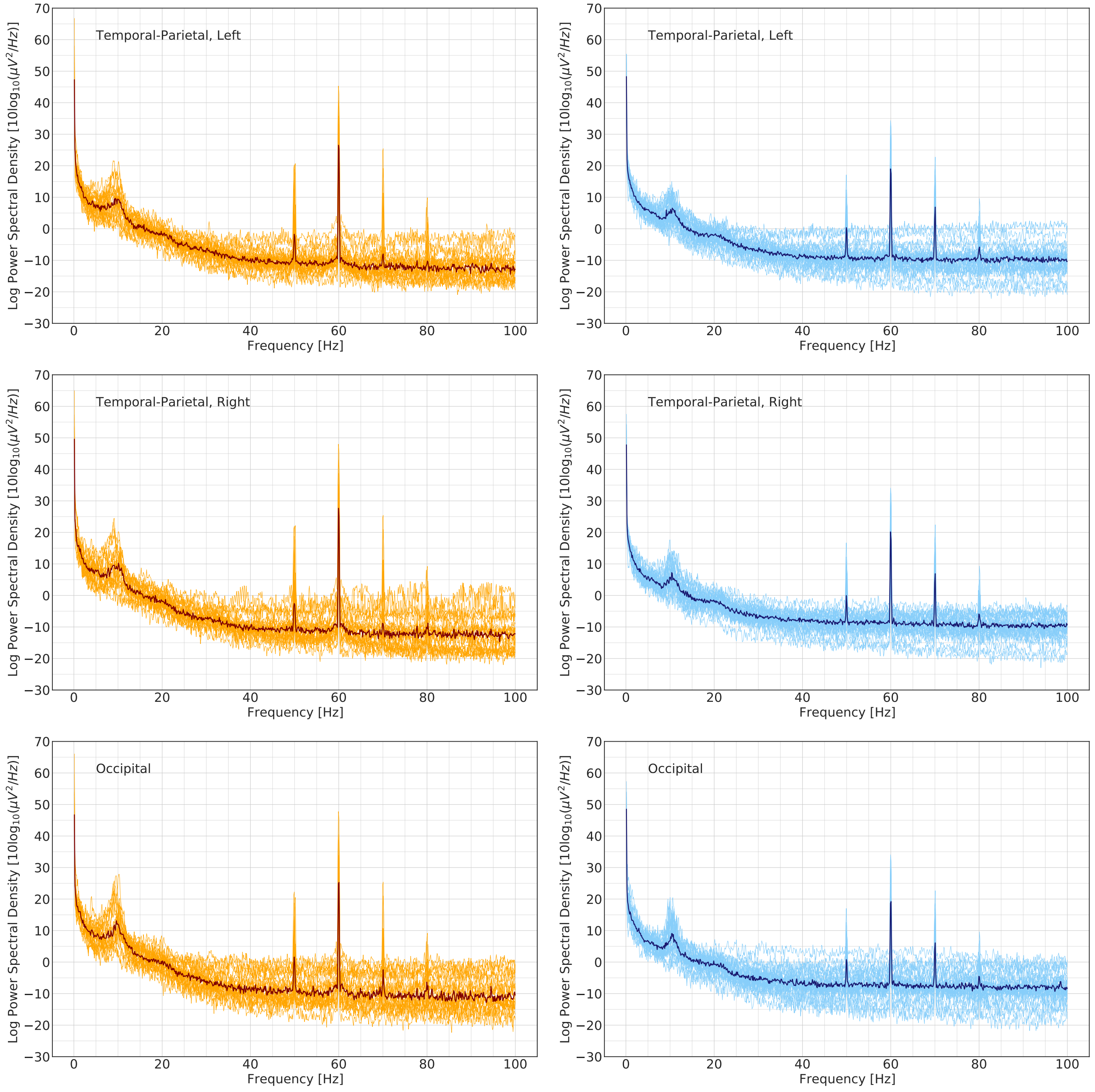

References

1. MATLAB. *9.7.0.1190202 (R2020a)* (The MathWorks Inc., Natick, Massachusetts, 2020).
2. Balkan, O., Virji-Babul, N., Miyakoshi, M., Makeig, S. & Garudadri, H. Source-domain spectral eeg analysis of sports-related concussion via measure projection analysis. In *2015 37th Annual International Conference of the IEEE Engineering in Medicine and Biology Society (EMBC)*, 4053–4056, DOI: 10.1109/EMBC.2015.7319284 (2015).
3. Dunkley, B. *et al.* Low-frequency connectivity is associated with mild traumatic brain injury. *NeuroImage: Clin.* **7**, 611–621 (2015).
4. Huang, M.-X. *et al.* Marked increases in resting-state meg gamma-band activity in combat-related mild traumatic brain injury. *Cereb. Cortex* **30**, 283–295 (2020).
5. Huang, M.-X. *et al.* Resting-state magnetoencephalography source imaging pilot study in children with mild traumatic brain injury. *J. Neurotrauma* **37**, 994–1001 (2020).
6. Nottage, J. F. & Horder, J. State-of-the-art analysis of high-frequency (gamma range) electroencephalography in humans. *Neuropsychobiology* **72**, 219–228 (2015).
7. Shackman, A. J. *et al.* Electromyogenic artifacts and electroencephalographic inferences. *Brain Topogr.* **22**, 7–12 (2009).
8. Hunter, J. D. Matplotlib: A 2d graphics environment. *Comput. Sci. & Eng.* **9**, 90–95, DOI: 10.1109/MCSE.2007.55 (2007).
